# Optimization of Marinating Process and Evaluation of Storage Stability in Bovine By-products

**DOI:** 10.3390/foods14173036

**Published:** 2025-08-29

**Authors:** Yuling Qu, Dan Deng, Li Zhang

**Affiliations:** College of Food Science and Engineering, Gansu Agricultural University, Lanzhou 730070, China; quyuling0106@163.com (Y.Q.); 924166027@163.com (D.D.)

**Keywords:** bovine by-products, marinating process optimization, storge stability, natural antioxidants, product quality

## Abstract

Given the demand for sustainable food solutions in China and the underutilization of bovine by-products, this study aimed to optimize the marinating process of bovine liver, heart, and rumen while evaluating their storage stability. An orthogonal experimental design was employed to systematically optimize the marinating agent ratio and incorporate natural antioxidants to inhibit lipid oxidation and microbial spoilage. Results demonstrated that the optimized marinating formula, which included 0.3 g/kg rosemary extract, exhibited optimal antioxidant and antimicrobial effects. This strategy not only slowed product pH decline but also improved product yield and texture, and significantly reduced thiobarbituric acid reactive substances (TBARS) values and carbonyl content (*p* < 0.05), while maintaining favorable sensory scores and extending shelf life. The study indicates that targeted marinating technology holds potential for transforming bovine by-products into high-value-added food products, offering innovative solutions to address both economic and environmental challenges and establishing a technical foundation for efficient by-product utilization and industrial upgrading.

## 1. Introduction

With the continuous growth of the global population and significant improvements in living standards, the demand for high-quality food is increasing. This trend has driven significant advancements in food-processing technologies. As an important livestock country, China produces a large amount of bovine by-products annually, which not only constitute an indispensable part of the livestock industry but also harbor enormous economic potential. According to data from the National Bureau of Statistics in 2023, China’s beef production reached 7.53 million tons, with a year-on-year growth rate of 4.8%. Bovine by-products, including but not limited to bovine liver, heart, and rumen, are rich in proteins, iron, and various vitamins and possess high nutritional and economic value. However, the utilization rate of bovine by-products remains relatively low, and many by-products are discarded or simply processed without full development, leading to significant resource waste and environmental impact [1]. In recent years, as public attention to healthy diets has increased, the efficient utilization of these by-products and the development of products that meet market demands and possess high added value have become major challenges faced by researchers and enterprises alike. Therefore, in-depth research on the processing techniques and storage characteristics of bovine by-products is of utmost importance for improving their utilization rate and promoting the sustainable development of the livestock industry in China.

Marinating is a traditional and widely used method of food preservation that plays a crucial role in meat processing. It not only extends the shelf life and imparts unique colors and enhances flavors by regulating the hydrolysis and oxidation processes of lipids in meat products [2], but also significantly affects a series of quality parameters, such as salt content, moisture content, water-holding capacity, water activity, and shear force [3,4]. However, the application of this technology to high-nutritional-value, high-water-activity bovine by-products (e.g., liver, heart, and rumen) faces significant limitations. On the one hand, previous studies primarily focused on muscle tissue [4,5], while the unique physicochemical properties of bovine by-products, characterized by high water activity and lipid content, pose specific challenges to conventional marinating techniques [2]. On the other hand, core issues such as the optimization of marinating agent formulations and the integration of natural antioxidants remain systematically unresolved. Although Kumar et al. [5] demonstrated that ginger and lemon extracts improved the water-holding capacity and reduced hardness in cured chicken, the efficient marination of bovine by-products still encounters bottlenecks like poor formulation adaptability and difficulty in controlling spoilage risks, hindering the full realization of their substantial economic potential.

These characteristics (high water activity and lipid content) render bovine by-products highly susceptible to quality deterioration during long-term storage, with lipid oxidation being the primary factor responsible for rancid off-odors, nutritional loss, and the potential formation of harmful compounds [6]. While minimizing exposure to oxygen and light remains crucial, the most effective mitigation strategy involves incorporating antioxidants, which are critical determinants for ensuring refrigerated storage stability and market competitiveness. Currently, the industry predominantly employs synthetic preservatives and antioxidants, such as butylated hydroxytoluene (BHT) and butylated hydroxyanisole (BHA) [7]. However, concerns regarding their safety have increased [8]. This has prompted researchers to search for natural-source antioxidants. Extensive research confirmed that plant extracts such as rosemary, cinnamon, nutmeg, and clove possess potent antioxidant and preservative capabilities, effectively delaying oxidation and extending the shelf life of meat products [9]. For instance, rosemary extract has been shown to outperform BHT in inhibiting protein and lipid oxidation in fresh pork patties, while simultaneously improving color stability, cooking yield, and textural properties [10]. Deflavored and decolorized rosemary extract containing 3% carnosic acid exhibited antioxidant efficacy comparable to that of BHA, with a synergistic effect observed between carnosol and carnosic acid at a 1:1 (*w*/*w*) ratio [11]. Edible gelatin-chitosan films incorporating Tunisian rosemary extract (0.5–2%) [12] or synergistic use with high-oxygen modified atmosphere packaging [13] significantly extended shelf life and improved stability. A 0.05% nutmeg extract was more effective than TBHQ in inhibiting the formation of mutagenic and carcinogenic heterocyclic amines (HCAs) in roasted beef patties and showed better oxidative stability during refrigerated storage [14]. Dipping or coating with 1% nutmeg essential oil significantly inhibited lipid oxidation and microbial growth in chicken fillets, extended their shelf life, and maintained their sensory attributes [15]. Incorporating a high concentration of cinnamon powder (2 g/100 g) during burger preparation enhanced antioxidant and antimicrobial activities, reduced lipid oxidation, improved sensory characteristics, and extended the storage period of burgers [16]. Cinnamon oil (0.025–0.05%) significantly enhanced the preservative efficacy of lamb meat during refrigeration [17]. Clove extract (0.1%) also significantly reduced oxidation indicators (TBARS and carbonyl content) in refrigerated beef patties [14,18]. These findings provide a robust foundation for the use of natural plant extracts as alternatives to synthetic antioxidants.

Given the limitations of bovine by-product marinating technology and the demonstrated potential of natural antioxidants, this study employed an L9 (3^4^) orthogonal experimental design to determine optimal marinating ratios with minimal experimental runs, overcoming the limitations of conventional single-factor experiments [3]. Concurrently, natural antioxidants, such as rosemary, cinnamon, and nutmeg extracts, have been integrated into the basic marinating preparation [8,19] to jointly inhibit lipid oxidation, microbial proliferation, and protein degradation [10,15], and construct a multifunctional preservation system in line with the trend of clean labels. 

Therefore, this study aims to explore the optimal marinating methods for bovine liver, heart, and rumen as primary materials based on orthogonal optimization and natural antioxidant integration, thereby providing a theoretical foundation for the innovative development of bovine by-products. We also evaluate the storage stability of the marinated products to provide scientific evidence for their industrial production and marketing. The expected outcomes of this research will not only open new avenues for the efficient utilization of bovine by-products but also drive technological innovation in China’s food industry, better meet consumers’ demands for healthy and convenient foods, and demonstrate significant economic and social benefits.

## 2. Materials and Methods

### 2.1. Sample Preparation

Fresh bovine by-products (liver, heart, and rumen) were supplied by Kangmei Modern Farming and Animal Husbandry Group Co., Ltd. (Linxia, China). Immediately after slaughter, the organs were removed and washed with water to remove the surface fascia and other impurities. After draining, samples were placed in polyethylene bags and transported to the laboratory on ice (0–4 °C). Rosemary extract was purchased from Henan Senyuan Botanical Natural Products Co., Ltd. (Xuchang, China), cinnamon extract from Shaanxi Junkangda Biotechnology Co., Ltd. (Xi’an, China), nutmeg extract from Shaanxi Haiyisi Biotechnology Co., Ltd. (Xi’an, China), and clove extract from Shaanxi Xi’an Virgin Biotechnology Co., Ltd (Xi’an, China). Freshness-preserving film and basic marinating agents were sourced from the local Beijing Hualian Supermarket; among the chemical reagents used were analytical grade.

### 2.2. Experimental Design for Optimization of Base Marinating Formulation

To determine the optimal basic marinating formulation ratios for bovine liver, heart, and rumen, an L9 (3^4^) orthogonal array design was employed to systematically examine the effects of the quantities of salt, sugar, monosodium glutamate, cooking wine, soy sauce, onion, ginger powder, and pepper powder on marinating efficacy. The experimental procedure comprised: (1) thorough manual mixing of bovine liver, heart, and rumen cubes (2 × 2 × 1 cm) with marinating mixtures according to weight percentages (Table S1); (2) placement of samples in trays, covered with food-grade plastic wrap; (3) static marination at 0–4 °C for 30 min (bovine liver), 45 min (bovine heart), and 60 min (bovine rumen). Sensory evaluation was used to assess the effects of marination. Initially, single-factor experiments were carried out to investigate the impact of the amount of each component added on the marinating effects, with each parameter tested in triplicate. Subsequently, orthogonal array experiments were conducted to refine the optimal marinating formulations based on preliminary single-factor results. The exact ratio of bovine by-products to marinating agents, along with detailed information on the ranges and levels of the independent variables and their corresponding coded values, is provided in Table S1.

### 2.3. Comparative Analysis of Oxidative Properties of Natural Antioxidants

#### 2.3.1. ABTS Cation Radical Scavenging Activity

The total antioxidant activity was determined using the ABTS^+^ radical cation discoloration assay [19]. ABTS solution (7 mM) was prepared using distilled water, and ABTS^+^ was produced by reacting with 2.45 mM K_2_S_2_O_8_. The resulting mixture was then placed in the dark at room temperature (~25 °C). After 12 h, the absorbance at 734 nm was adjusted to 0.70 ± 0.05 by adding absolute ethanol. ABTS + solution (4 mL) was added to 40 μL of the sample solution, mixed thoroughly, and the absorbance (AS) was measured at 734 nm after 30 min using phosphate buffer as a control (AB). ABTS scavenging activity was calculated using the following equation:

$${\mathrm{ABTS}}^{+}•\;\mathrm{scavenging}\;\mathrm{rate}/\left(\%\right)=\frac{\mathrm{AB}-\mathrm{AS}}{\mathrm{AB}}\times 100\%$$

#### 2.3.2. DPPH Free Radical Scavenging Activity

DPPH free radical scavenging capacity was determined using the change in absorbance of purple-colored DPPH solution (0.6 mM) upon reaction with antioxidants measured at 517 nm [20]. A DPPH solution was prepared at a concentration of 0.2 mmol/L in 95% ethanol. Subsequently, 0.5 mL of the sample solution was mixed thoroughly with 3.5 mL of the DPPH solution and allowed to react for 30 min in the dark. Finally, absorbance was measured at 517 nm. The free radical scavenging rate of DPPH was calculated using the following equation:

$$\mathrm{Free}\;\mathrm{radical}\;\mathrm{scavenging}\;\mathrm{rate}\;\mathrm{of}\;\mathrm{DPPH}/\left(\%\right)=\frac{A_{i}-A_{j}}{A_{0}}\times 100\%$$

where *A_i_* represents the absorbance of the sample solution + DPPH solution, *A_j_* represents the absorbance of the sample solution + 95% ethanol solution, and *A*_0_ represents the absorbance of the 95% ethanol solution + DPPH solution.

#### 2.3.3. Reducing Power

Reductive power assays are commonly used to assess the ability of natural antioxidants to provide electrons or hydrogen, which is positively correlated with antioxidant activity [21]. The sample (1 mL) was mixed with 2.5 mL phosphate buffer and 2.5 mL of 1% K_3_Fe(CN)_6_ and allowed to react in the dark for 20 min. Then, 2.5 mL of 10% trichloroacetic acid was added to terminate the reaction. The resultant mixture was centrifuged at 3000 rpm for 10 min. The supernatant obtained (2.5 mL) was treated with 0.5 mL of 0.1% FeCl_3_ and 2.5 mL of distilled water. Finally, the absorbance of the reaction mixture was determined at 700 nm.

### 2.4. Optimization of the Amount of Natural Antioxidant

Following the establishment of the optimal base-marinating formulations for bovine liver, heart, and rumen, gradient concentrations (0.06, 0.12, 0.18, 0.24, and 0.30 g/kg) of food-grade powdered natural antioxidants (rosemary, cinnamon, nutmeg, and clove extracts) were incorporated into each substrate. TBARS value served as the primary evaluation metric for dose-response analysis to systematically determine the optimal incorporation levels of each natural antioxidant within respective bovine by-products.

### 2.5. Determination of Nutritional Components

The moisture content was determined using the direct drying method described in GB 5009.3-2016 [22]. The ash content was evaluated using the scorch weighing method in GB 5009.4-2016 [23]. The crude protein content was determined using the Kjeldahl method with reference to GB 5009.5-2016 [24]. The crude fat content was measured according to the Soxhlet extraction method described in GB 5009.6-2016 [25].

### 2.6. Evaluation of Storage Stability of Bovine By-products During Storage

Refrigerated bovine by-products underwent organ-specific sampling regimens after marination and storage at 0–4 °C. Bovine liver samples were collected on Days 0, 3, 7, 9, 10, 11, and 12 of refrigeration, while bovine heart and rumen specimens were collected on Days 0, 3, 7, 9, 11, 13, and 15. A comprehensive analysis of all samples included: (1) edible quality (including colorimetric properties, pH, sensory scores, textural profile analysis, and processing yield; (2) oxidative stability parameters (TBARS values and protein carbonyl content); and (3) microbiological safety indices.

#### 2.6.1. Edible Quality

##### Color Measurement

For each treated sample, the surface color was measured using a hand-held colori meter (CR—400; Konica Minolta, Tokyo, Japan). Color coordinates ranged from L = 0 (black) to L = 100 (white), −a (greenness) to +a (redness), and −b (blueness) to +b (yellowness). Each sample was tested six times, and the L*, a*, and b* values were recorded and averaged.

##### pH

The pH was determined using a portable pH meter (SenvenGo, MettlerToledo, Greifensee, Switzerland) with a penetrating electrode. The pH probe was calibrated using two buffers of pH 4.0 and 7.0, which were maintained at 4 °C. Each sample was measured in triplicate.

##### Sensory Evaluation

Sensory analysis was conducted on treated bovine by-products. This analysis strictly adhered to the Chinese National Standard: Criterion for sensory evaluation of meat and meat products (GB/T 22210-2008) [26]. Sensory evaluation was performed by a trained panel of 10 assessors (5 males and 5 females; aged 20–40 years). The samples were assessed for five key sensory attributes: texture, organizational structure, color, flavor, and overall acceptability. The specific evaluation method was as follows: Samples were placed in 10 mL sensory cups. Assessors evaluated the samples through visual examination, touch using chopsticks, and smell to describe and score the color, texture, and organizational structure, odor, and overall acceptability. Sensory scores were categorized into three levels: 0–7 points, 8–14 points, and 15–20 points. The detailed scoring criteria for each attribute are listed in Table 1. The final sensory score represents the average of all panelists’ ratings.

##### Texture Properties

The marinated bovine by-products were cut into 1 cm × 1 cm × 1 cm pieces for textural characterization. Texture properties were determined according to the method described by Tan et al. [27] with slight modifications. The program of the texture analyzer TA.XT Express C (Stable Micro Systems, Godalming, Surrey, UK) was set to TPA mode. The probe model P/36R was used with a trigger force of 5.0 g, displacement of 15 mm, pre-test velocity of 2.0 mm/s, test velocity of 1.0 mm/s, and a post-test velocity of 10.0 mm/s. The time interval between two compressions was 5.0 s with 50% compression for the test. The indices of hardness, elasticity, chewability, recovery, and cohesion were selected and measured on six samples, and the final results were expressed as the average of the six samples.

#### 2.6.2. Processing Yield of Pre-Prepared Bovine By-products

The procedure commenced by thoroughly removing excess moisture from the surface of the marinated bovine by-products using absorbent paper and then accurately measuring their initial weight (*W*_1_). Subsequently, the samples were roasted at predetermined temperatures and times. Following completion of roasting, the samples were removed and allowed to cool naturally to room temperature (~25 °C). The final weight (*W*_2_) was recorded using a precise weighing scale. The processing yield of the pre-prepared grilled bovine by-products was calculated using the following formula:

$$\mathrm{processing}\;\mathrm{yield}\;\left(\%\right)=\frac{W_{2}}{W_{1}}\times 100\%$$

#### 2.6.3. Oxidative Property

##### Determination of TBARS Value

TBARS is an indicator used to detect the degree of deterioration in samples, assessing the level of lipid oxidation. A higher value indicates a greater degree of oxidation, which can compromise the quality and stability of food products. The TBARS values were determined according to the method described by Tan et al. [27], with slight modifications. Briefly, 10 g of the sample was added to 25 mL of distilled water, homogenized, and 25 mL of 5% (*v*/*v*) trichloroacetic acid was added. After thorough mixing, the sample was allowed to stand for 30 min. The supernatant (5 mL) was mixed with 5 mL of 0.02 mol/L thiobarbituric acid, heated in a constant temperature water bath at 80 °C for 40 min, and immediately cooled to room temperature (~25 °C). The absorbance was measured at 532 nm, and the TBARS value was expressed as malondialdehyde (MDA) mg/kg of the bovine by-product.

##### Determination of Carbonyl Content

The carbonyl content of the bovine by-products was measured using 2,4-dinitrophenylhydrazine (DNPH) according to the method described by Tan et al. [27] with slight modifications. Briefly, 0.2 g of the sample was added to 2 mL of 20 mmol/L phosphate buffer (containing 0.6 mol/L NaCl, pH 6.5), homogenized well, and then centrifuged at 4 °C, 8000 rpm for 10 min. A total of 0.32 mL of the supernatant was placed in two 10 mL tubes as the control and assay tubes. Subsequently, 0.24 mL of DNPH solution (containing 2 mol/L HCl) was added to the control tube, and 0.24 mL of 2 mol/L HCl was added to the assay tube. After mixing, the tubes were incubated at 37 °C for 1 h, with shaking every 10 min. Subsequently, 0.15 mL of 20% trichloroacetic acid solution was added to the tube, allowed to stand for 5 min, and centrifuged at 4 °C and 12,000 rpm for 15 min. The precipitate was washed three times with 0.6 mL of ethyl acetate-ethanol solution (1:1, *v*/*v*) to remove untreated DNPH and then centrifuged at 4 °C, 12,000 rpm for 10 min. Finally, 0.2 mL of guanidine hydrochloride was added to dissolve the precipitate. After all the precipitate was dissolved, it was centrifuged at 4 °C, 12,000 rpm for 15 min, and 200 μL of the supernatant was added to measure the absorbance at 370 nm. The carbonyl content of the protein was calculated as follows:

$$carbonyl\;content/\left(\mu mol/g\right)=\frac{A_{assay}-A_{control}}{8\times W}$$

where *W* is the sample mass (g).

#### 2.6.4. Microbial Analysis

The total viable count (TVC) of the sample was quantified using plate count agar according to Chinese standard GB 4789.2-2022 [28], and the results of the TVC were expressed as lgCFU/g of sample. Specifically, 10 g of the sample was weighed in a sterilized homogenization cup containing 90 mL of sterile saline solution. The mixture was then homogenized at 10,000 rpm for 1 min. Subsequently, serial dilutions were prepared using sterile saline solution, and triplicate 1 mL samples of the appropriate dilutions were transferred onto selective agar plates. TVC was determined using Plate Count Agar (PCA) after incubation at 37 °C for 48 h.

### 2.7. Statistical Analysis

Origin 2021 (OriginLab Corp., Northampton, MA, USA) was used for data sorting and image processing. SPSS software (v.26.0, IBM, Chicago, IL, USA) was used for the analysis of variance (ANOVA), and Duncan’s multiple range test was employed to determine differences (significance was defined at *p* < 0.05).

## 3. Results and Discussion

### 3.1. Analysis of Experimental Results from Basic Marinating Studies

Marinating agents are key determinants of the fundamental flavor of food, and different types of marinating agents can significantly influence the flavor, color, texture characteristics, storage duration, and sensory quality of food. Based on the single-factor experimental results (Figure S1), an orthogonal array design (L9) was selected to systematically evaluate the interactions among multiple marinating agents, as it allows for the efficient identification of dominant factors with minimal experimental runs [29]. Similar approaches have been successfully applied in meat processing studies to optimize complex formulations. For instance, Hao et al. [3] utilized an L16 orthogonal array to determine the optimal salt and phosphate ratios in cured pork, demonstrating the robustness of the method in balancing sensory and functional properties. For bovine liver, the analysis of *R* values revealed that the impact of each component on the marinating effect was as follows: NaCl (A) > onion (H) > sugar (B) > cooking wine (F) > ginger powder (E) > pepper powder (D) > monosodium glutamate (C) > soy sauce (G). When the optimal combination of marinating agents is A_2_H_2_B_2_F_2_E_2_D_2_C_2_G_2_, i.e., NaCl 1.0%, sugar 0.6%, monosodium glutamate 0.3%, pepper powder 0.25%, ginger powder 0.15%, cooking wine 2.4%, soy sauce 2.0%, and green onion 3%, the sensory score of marinated bovine liver reaches its highest value of 88.70 (Table S3). Variance analysis showed that salt, cooking wine, and green onion had a significant impact on the marinating effect of bovine liver (*p* < 0.05) (Table S4). To verify the effectiveness of this formula, three parallel marinating experiments on bovine liver were conducted, and the final sensory score reached 90.5, proving the reliability of the experimental results.

For bovine heart, based on the *R* value analysis, the order of influence of each component on the marinating effect was: onion (H) > sugar (B) > NaCl (A) > ginger powder (E) > pepper powder (D) > cooking wine (F) > soy sauce (G) > monosodium glutamate (C). When the optimal combination of marinating agents was H_2_B_2_A_2_E_2_D_2_F_2_G_2_C_2_, i.e., salt 0.8%, sugar 0.6%, monosodium glutamate 0.4%, pepper powder 0.2%, ginger powder 0.15%, cooking wine 2%, soy sauce 2.4%, and green onion 4%, the sensory score of marinated bovine heart reaches its highest value of 93.00 (Table S5). Variance analysis further confirmed that green onion has a significant impact on the marinating effect of the bovine heart (*p* < 0.05) (Table S6). Similarly, to verify the effectiveness of this formula, three parallel marinating experiments of bovine heart were conducted, and the final sensory score reached 91.50, indicating the reliability of the experimental results.

For the bovine rumen, according to the *R* value analysis, the degree of influence of each component on the marinating effect was as follows: NaCl (A) > ginger powder (E) > onion (H) > sugar (B) > monosodium glutamate (C) > pepper powder (D) > soy sauce (G) > cooking wine (F). When the optimal combination of marinating agents was A_2_E_2_H_2_B_2_C_2_D_2_G_2_F_2_, i.e., NaCl 1.0%, sugar 0.5%, monosodium glutamate 0.3%, pepper powder 0.2%, ginger powder 0.15%, cooking wine 2.4%, soy sauce 1.6%, and green onion 4%, the sensory score of marinated rumen reaches its highest value of 93.20 (Table S7). The results of variance analysis showed that salt and ginger powder had a significant impact on the marinating effect of the bovine rumen (*p* < 0.05) (Table S8). To verify the effectiveness of this formula, three parallel marinating experiments of the rumen were conducted, and the final sensory score reached 93.20, further confirming the reliability of the experimental results.

#### 3.1.1. Optimization of Marinating Formulas Incorporating Natural Antioxidants

##### Antioxidant Activities of Natural Antioxidants

**Figure 1:**
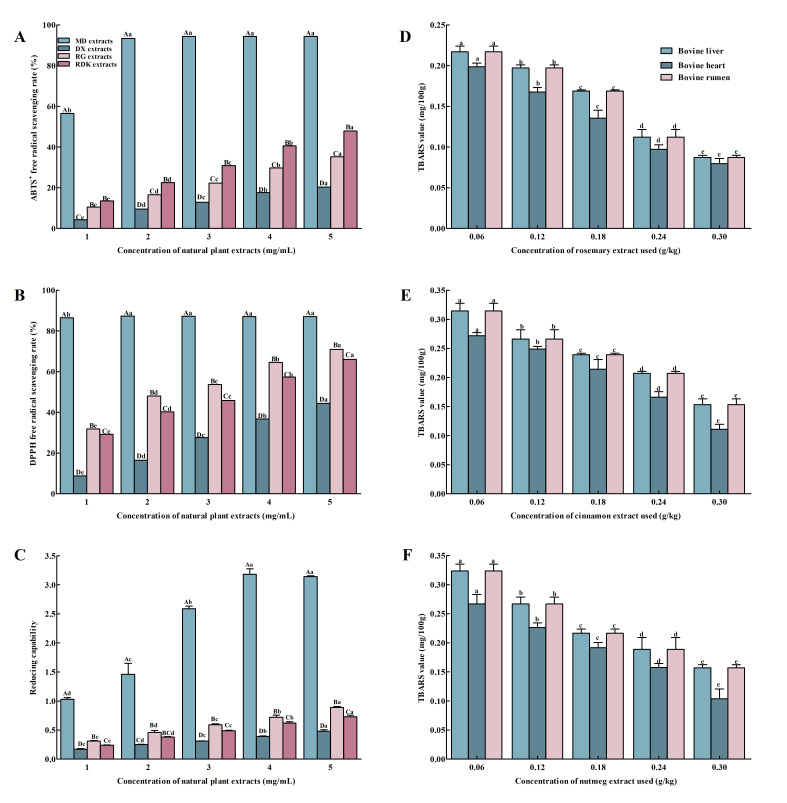
In vitro antioxidant properties of natural plant extracts and single-factor optimization of their addition levels in bovine by-products. (**A**–**C**) concentration-dependent effects on radical-scavenging capacity: MD, basic marinating + rosemary extract; RG, basic marinating + cinnamon extract; RDK, basic marinating + nutmeg extract. (**A**) ABTS^+^• radical-scavenging activity, (**B**) DPPH• radical-scavenging activity, (**C**) reducing power. (**D**–**F**) single-factor optimization trials to determine the optimal concentration of bovine by-products: (**D**) rosemary extract, (**E**) cinnamon extract, and (**F**) nutmeg extract. Data are presented as mean ± SD (*n* = 3). Different lowercase letters indicate significant differences within groups (*p* < 0.05), and different uppercase letters indicate significant differences between groups (*p* < 0.05).

The ABTS^+^ radical scavenging method was used to evaluate the antioxidant capacity of multi-component mixtures, comprehensively reflecting the overall antioxidant level. In this method, the ABTS^+^ radical reacts with antioxidant substances, neutralizing cations and causing a linear change in absorbance [30]. As shown in Figure 1A, the order of strength of the scavenging rate of ABTS^+^ radicals by the four natural plant extracts was as follows: rosemary extract > nutmeg extract > cinnamon extract > clove extract. The scavenging rates of nutmeg, cinnamon, and clove extracts against ABTS^+^ radicals increased with increasing concentration. When the concentration of rosemary extract reaches 2 mg/mL, its inhibition rate against ABTS^+^ radicals reached 93.35%, and as the concentration further increases, the growth trend of the ABTS^+^ radical scavenging rate gradually levels off. When the concentrations of rosemary, clove, cinnamon, and nutmeg extracts were all 5 mg/mL, their scavenging rates against ABTS^+^ radicals were 94.34%, 20.34%, 35.16%, and 47.91%, respectively. This indicated that the scavenging ability of rosemary extract against ABTS^+^ radicals is the strongest, while that of clove extract is the weakest.

The DPPH method, known for its high accuracy, is widely used to measure in vitro antioxidant activity. DPPH reacts with ethanol to form a deep purple compound; when an antioxidant is added, it pairs with the lone pair of electrons on the DPPH molecule, causing the color to fade. By observing this color change, the ability of the substance to scavenge DPPH radicals can be assessed [31]. According to Figure 1B, the order of scavenging ability of DPPH radicals by rosemary, clove, cinnamon, and nutmeg extracts was as follows: rosemary extract > cinnamon extract > nutmeg extract > clove extract. The scavenging rates of DPPH radicals by clove, cinnamon, and nutmeg extracts increased with increasing concentration, whereas the performance of rosemary extract was similar to that of ABTS^+^ radical scavenging rate, reaching the highest scavenging rate within the concentration range of 1.0–2.0 mg/mL. Even if the concentration continued to increase, there was no significant difference in the scavenging rate of DPPH radicals by the rosemary extract (*p* > 0.05).

The antioxidant activity of substances can also be assessed through their reducing power, where typically, the higher the reducing power, the more pronounced the antioxidant effect [32]. As shown in Figure 1C, the reducing power of all four natural plant extracts increased with increasing concentration; however, at the same concentration, the reducing power of rosemary extract was significantly higher than that of the other three plant extracts, while the reducing power of clove extract was the lowest. Although the principles of the three antioxidant capacity test methods were different, they showed a higher antioxidant capacity regardless of which test method was used, while the other showed the lowest antioxidant capacity. Therefore, clove plant extract was not used in subsequent experiments.

##### Single-Factor Analysis of Natural Antioxidants

Lipid oxidation is initiated by free radical chain reactions, which generate numerous compounds that affect the sensory properties of food, leading to a decline in meat and meat product quality. In particular, polyunsaturated fatty acids (PUFAs) in lipids are more susceptible to free radical attacks due to their structural characteristics, resulting in off-flavors. To inhibit lipid oxidation, many antioxidants have been employed as additives or supplements in meat or meat products. The TBARS value is a critical indicator of the formation of secondary oxidation products, such as MDA, acrolein, and conjugated dienes, during lipid oxidation [33,34,35]. As shown in Figure 1D–F, as the amount of rosemary, cinnamon, and nutmeg extract increases, the TBARS values of bovine liver, heart, and rumen showed a gradually decreasing trend. When the amount of these extracts was set to 0.3 g/kg, the TBARS values of bovine by-products were significantly lower than those of the other treatment groups (*p* < 0.05). According to the National Food Safety Standard for the Use of Food Additives (GB 2760-2024) [36], the maximum allowable addition amounts of rosemary, cinnamon, and nutmeg extracts in meat products were 0.3 g/kg. Therefore, to effectively inhibit lipid oxidation and ensure product quality, it was recommended to add rosemary, cinnamon, and nutmeg extracts at a concentration of 0.3 g/kg to bovine liver, heart, and rumen.

### 3.2. Nutritional Components of Marinated Bovine By-products

According to Table 2, during the marinating process, the fat, moisture, and ash content of bovine by-products (including bovine liver, heart, and rumen) did not show significant differences compared to the fresh group, indicating that marinating had little effect on these components. However, the situation is different for protein content. Specifically, the protein content of bovine liver and heart significantly decreased after marinating treatment, showing a notable difference compared to the fresh bovine by-products that had not undergone marinating. Additionally, the protein content in the rumen was significantly decreased after marination with nutmeg extract. This reduction in protein content may be attributed to two factors: first, the salt used during the marinating process may reduce protein solubility, leading to the extraction of some proteins and consequently lowering the detectable protein content; second, the antioxidant extracts added may contain enzymes that can affect the hydrolysis of proteins, resulting in a decrease in protein content.

### 3.3. Effect of Refrigeration on Edible Quality

#### 3.3.1. Color Analysis

**Figure 2:**
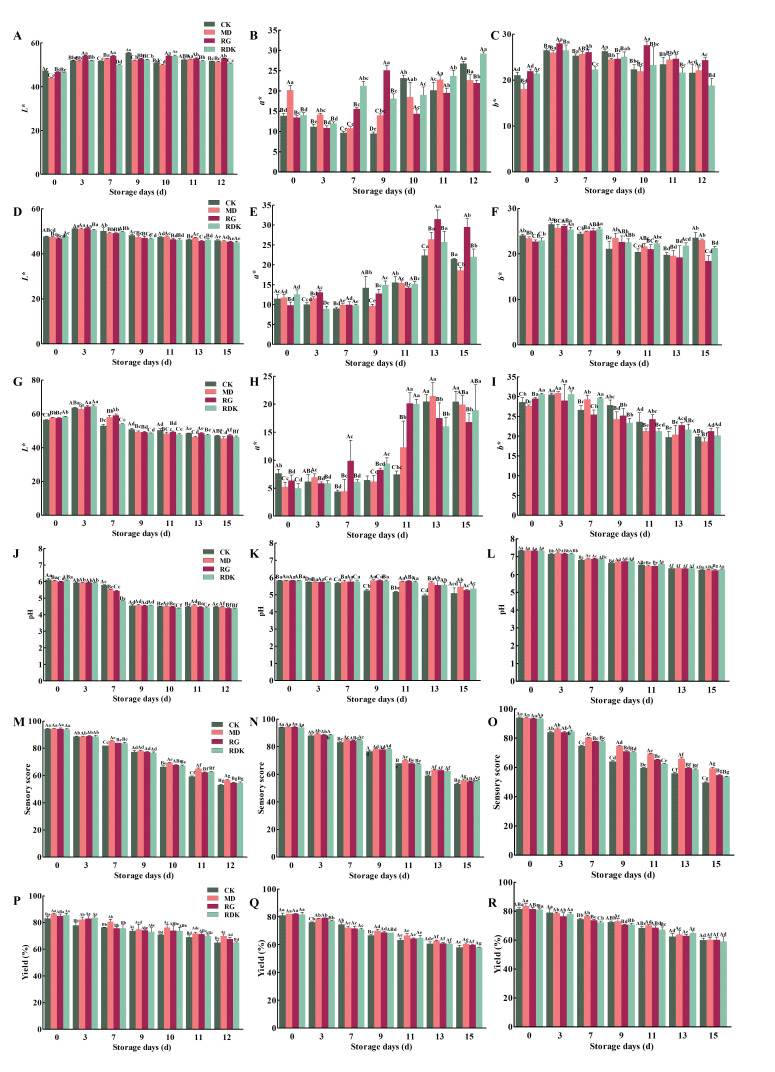
Effect of refrigerated storage time on chromaticity, pH, sensory evaluation scores, and processing yield of bovine by-products (liver, heart, rumen). (**A**–**C**) chromaticity changes in the bovine liver, (**D**–**F**) chromaticity changes in the bovine heart, (**G**–**I**) chromaticity changes in the bovine rumen. (**J**–**L**) correspond to the pH measurement results for the bovine liver, heart, and rumen, respectively. (**M**–**O**) correspond to the sensory evaluation scores for the bovine liver, heart, and rumen, respectively. (**P**–**R**) correspond to the processing yield results for bovine liver, heart, and rumen, respectively. Data are expressed as mean ± standard deviation (*n* = 3). Different lowercase letters denote significant differences within groups (*p* < 0.05), and different uppercase letters denote significant differences between groups (*p* < 0.05).

Color is a crucial indicator for evaluating meat quality and a key factor influencing consumer purchasing decisions [37]. Better-colored meat products can enhance consumer purchase intentions and boost sales. During the oxidation of bovine by-products, the formation of carbonyl and alcohol compounds (e.g., hexanol, pentanol, and cholesterol) leads to changes in color. Meat color is primarily determined by metmyoglobin content in muscle [38]. Myoglobin is transformed into oxymyoglobin (light pink color), which results in brighter red meat, and upon oxidation, it is converted into metmyoglobin during storage. The changes in lightness (L*), redness (a*), and yellowness (b*) values of the refrigerated bovine by-products under different treatments were comprehensively presented in Figure 2. The L* values of all groups of bovine by-products exhibited an initial increase, followed by a decrease as the storage time increases. In the early stages of storage (0–3 days), the L* value significantly increased (*p* < 0.05), primarily due to changes in the pigment protein structure and internal moisture exudation [39,40]. However, after 7 days of storage, the L* values of most samples started to decline, consistent with the findings of Sujiwo et al. [41] regarding the trend of L* value changed in refrigerated chicken breast meat. As storage time increases, myoglobin in bovine by-products undergoes oxidation, accompanied by a series of biochemical reactions that produce pigmented substances, leading to a decrease in L* value [42]. The trend of b* value changes was similar to that of L* values, also showing an initial increase followed by a decrease. This may be due to fat oxidation, which causes the color of bovine by-products to shift from red to yellow, resulting in an increase in the b* value, or it could be influenced by microbial activity [43]. Notably, the variation in b* values was relatively smaller in bovine liver and heart products. The a* value was closely related to the content of myoglobin pigments and the valence state of iron ions they contain. During storage, myoglobin in bovine by-products oxidizes to metmyoglobin, leading to an increase in the a* value [44,45]. In the refrigeration process of pre-prepared bovine by-products, the a* value showed an upward trend, particularly after 11 days of storage, where the a* value of the control group was significantly lower than that of the other three groups treated with antioxidants (*p* < 0.05). This is likely due to the presence of antioxidants, which slow down the oxidation of myoglobin, thereby delaying the color change of the meat samples. These results are similar to those of the inhibition of lipid and protein oxidation in raw ground pork by Terminalia arjuna fruit extract during refrigerated storage [42].

#### 3.3.2. pH Analysis

The pH value is closely associated with the freshness, color, tenderness, and other factors of meat products, serving as a crucial indicator for assessing meat quality. As shown (Figure 2J–L), the pH values of all bovine by-products exhibited a continuous downward trend during cold storage. For bovine liver and heart products, a significant decrease in pH mainly occurred during the middle to late stages of cold storage (*p* < 0.05). In the case of rumen products, a significant decrease in pH was already noted during the early stage of cold storage (*p* < 0.05). The decline in pH during storage may be attributed to lipid oxidation-driven processes: (1) release of acidic enzymes from lysosomal rupture, (2) accumulation of short-chain organic acids (e.g., formic acid and acetic acid) from fatty acid breakdown, and (3) microbial acid production potentiated by oxidative damage. In the later stages of cold storage, the pH values of pre-prepared bovine heart products marinated with rosemary, cinnamon, or nutmeg extracts were significantly higher than those of the control group without added antioxidants, further confirming that rosemary, cinnamon, and nutmeg extracts possess certain preservative effects.

#### 3.3.3. Sensory Score Analysis

Sensory scores were obtained by evaluating the color, texture, flavor, tissue condition, and overall acceptability of pre-prepared bovine by-products (Figure 2M–O). The results showed that the sensory scores of the basic marinade group and the groups treated with rosemary extract, cinnamon extract, and nutmeg extract significantly decreased over the extended period of cold storage (*p* < 0.05). In the later stages of cold storage, the sensory scores of products marinated with rosemary, cinnamon, and nutmeg extracts were significantly higher than those treated only with the basic marinade (*p* < 0.05). These three groups exhibited a more uniform color, better texture elasticity and formability, and less gamey odor. These findings are consistent with the texture analysis results. These results indicate that rosemary, cinnamon, and nutmeg extracts can effectively prevent the deterioration of sensory quality in marinated bovine by-products during cold storage. In particular, during the cold storage of pre-prepared grilled rumen products, those marinated with rosemary extract showed excellent sensory scores. According to reports, rosemary extract possesses strong antioxidant capabilities and inhibits microbial growth, which can significantly delay the degradation of animal product quality during cold storage [46].

#### 3.3.4. Product Yield Analysis

The yield of meat products is significantly influenced by the juiciness and processing characteristics of the meat, and the extent of yield loss can serve as an indicator of the water-holding capacity of the product [47]. A primary factor contributing to yield reduction is the denaturation of myofibrillar proteins induced by marination and storage, leading to the loss of exudates containing soluble proteins and consequently decreased product yield [48,49]. During refrigerated storage, the yield of pre-prepared grilled bovine liver, heart, and rumen exhibited a significant decline. However, compared to the control group, the treated groups showed a significant improvement in the yield of bovine by-products, and this advantage remained evident even after 15 days of refrigerated storage (Figure 2P–R). Research has indicated that the addition of binders, such as dry potato extract, directly enhances product water-holding capacity, thereby increasing yield [50]. Furthermore, incorporating plant extracts (e.g., rosemary, cinnamon, and nutmeg) significantly improves the water-holding capacity of meat through their antioxidant properties, which contribute to enhanced juiciness and textural properties [51]. More critically, these extracts can promote the cross-linking of muscle proteins in pre-prepared bovine by-products, facilitating the formation of a stable three-dimensional network structure. This effectively reduces cooking losses, and ultimately enhances product yield [52].

#### 3.3.5. Texture Properties Analysis

**Figure 3:**
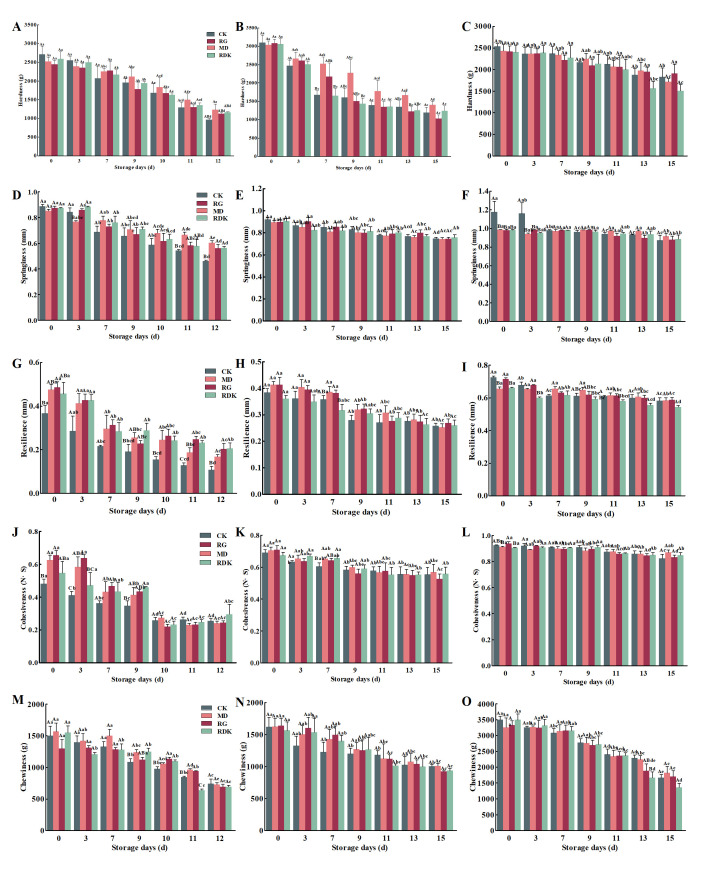
Effect of refrigerated storage time on textural properties (hardness, elasticity, chewiness, resilience, cohesiveness) of prepared bovine by-products (bovine liver, heart, rumen). The figure displays textural parameters grouped by bovine by-product: (**A**,**D**,**G**,**J**,**M**) textual properties of bovine liver; (**B**,**E**,**H**,**K**,**N**) textual properties of bovine heart; and (**C**,**F**,**I**,**L**,**O**) present for textual properties of bovine rumen, respectively. Data are expressed as mean ± standard deviation (*n* = 3). Different lowercase letters indicate significant differences within groups (*p* < 0.05), and different uppercase letters indicate significant differences between groups (*p* < 0.05).

Texture characteristics can be used to characterize the structure, tissue condition, and mouthfeel of meat and meat products, serving as critical indicators influencing consumer acceptance. As shown in Figure 3, the texture characteristics of the control group by-products declined most rapidly, indicating the fastest degradation in quality. With the extension of storage time, the hardness of all treatment groups showed a downward trend, primarily due to changes in protein structure and enhanced protease activity caused by protein oxidation [53]. Samples of by-products treated with rosemary and nutmeg extracts exhibited a significant increase in chewiness and cohesiveness values. Compared with the basic marinade treatment, the use of rosemary, cinnamon, and nutmeg extracts for marinating significantly improved the textural quality of pre-prepared bovine by-products during storage, specifically manifested by smaller changes in hardness, springiness, and elasticity. In meat products, the loss of chewiness is largely influenced by the hydrolysis of collagen and myofibrillar proteins [54]. The enhancement of chewiness and cohesiveness is associated with the fineness and integrity of the product, and the increase in these values may be due to the promotion of protein hydrolysis by the extracts, with the generated hydrolysates covering the surface of fat droplets, thereby enhancing gel strength [55]. In particular, treatment with rosemary extract can more effectively mitigate the loss of chewiness in pre-prepared grilled bovine by-products during storage.

### 3.4. Effect of Refrigeration on Oxidative Properties

#### 3.4.1. TBARS Values Analysis

**Figure 4:**
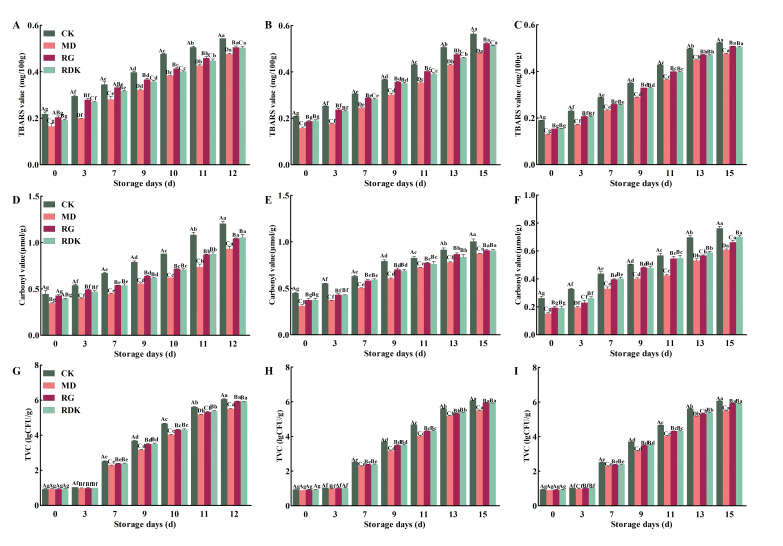
Effect of refrigerated storage time on lipid oxidation (TBARS values), protein oxidation (carbonyl content), and microbial proliferation (TVC) in bovine by-products (bovine liver, heart, and rumen). The figure groups data by analytical parameter: TBARS values ((**A**): bovine liver, (**B**): heart, (**C**): rumen); Carbonyl content ((**D**): bovine liver, (**E**): heart, (**F**): rumen); TVC ((**G**): bovine liver, (**H**): heart, (**I**): rumen). Data are presented as mean ± standard deviation (*n* = 3). Different lowercase letters denote significant differences within groups (*p* < 0.05), and different uppercase letters denote significant differences between groups (*p* < 0.05).

The TBARS values quantify aldehydes that are reactive with barbiturates, related to the accumulation of secondary products, and are widely used as an indicator of fat oxidation [56]. Fat oxidation involves a series of interactions between unsaturated fatty acids and molecular oxygen that produce labile primary products that can be further decomposed into the secondary product, MDA [56]. Higher MDA values indicate more severe fat oxidation and rancidity [45]. According to Karabagias et al. [57], meat products begin to develop unpleasant oxidized odors when the TBARS value in meat products reaches 0.5 mg/kg. As shown in Figure 4A–C, the TBARS values of the differently treated samples all showed a significant upward trend with the extension of storage time (*p* < 0.05). By day 11, the TBARS values of the control groups for bovine liver and heart reached 0.51 mg/kg; by day 15, the TBARS value of the control group for rumen reached 0.52 mg/kg, at which point the samples began to emit undesirable odors. In contrast, the TBARS values of the experimental groups remained below 0.5 mg/kg, indicating better quality. Despite the increasing degree of oxidation in all four sample groups during cold storage, the extent of oxidation in the experimental groups was consistently lower than that in the control groups, especially in the samples treated with rosemary extract, which demonstrated superior antioxidant effects (*p* < 0.05). This superiority originates from the unique phenolic diterpenes in rosemary extract (e.g., carnosic acid, rosmarinic acid, and carnosol), which disrupt the free radical chain reaction of lipid oxidation by donating hydrogen atoms (H•) and electrons [58,59,60]. The specific molecular mechanisms include: (1) free radical quenching, in which the phenolic hydroxyl group (—OH) reacts with lipid peroxyl radicals (LOO•) to form stable phenoxyl radicals (reaction: Phenolic-OH + LOO• → Phenolic-O• + LOOH), thereby halting the peroxidation of unsaturated fatty acids and consequently reducing MDA formation [61]; (2) synergistic metal chelation, in which carnosic acid effectively chelates pro-oxidant metal ions (e.g., Fe^2+^/Cu^2+^), inhibiting the Fenton reaction (Fe^2+^ + H_2_O_2_ → Fe^3+^ + OH• + OH^−^) that initiates lipid oxidation. In contrast, cinnamon and nutmeg extracts lack comparable metal-chelating capacity [62]. Studies conducted by Wang et al. [63] showed that flavonoid substances extracted from onion skin powder, which were added to premade beef patties as natural antioxidants, effectively prevented lipid oxidation.

#### 3.4.2. Carbonyl Content Analysis

The content of carbonyl in proteins is an important index for determining the degree of protein oxidation in meat and is also one of the key factors leading to the deterioration of quality in pre-prepared meat and meat products [64]. The increase in protein carbonyls shows that the muscle proteins were subjected to oxidative stress, which leads to the oxidative degradation of some amino acid side chains, such as lysine, proline, arginine, and histidine residues [65]. The higher the degree of protein bio-oxidation, the higher the carbonyl content. As shown in Figure 4D–F, the carbonyl content of all treatment group samples increased significantly with the extension of cold storage time (*p* < 0.05), primarily due to the intensified oxidation of myofibrillar proteins in bovine by-products and the reaction between lipid oxidation products and protein amines, which promotes indirect protein oxidation. These factors collectively contribute to the continuous increase in the carbonyl content of bovine by-products. The trend in carbonyl value changes was consistent with that of the TBARS values. The carbonyl values of the treatment group samples were significantly lower than those of the control group samples (*p* < 0.05), with the rosemary extract treatment group showing the best effect (*p* < 0.05). This may be because the phenolic compounds in rosemary can bind with proteins to form covalent compounds, preventing the formation of protein carbonyls and thereby effectively maintaining the quality of bovine by-products, given that protein carbonyls play a crucial role in protein oxidation. Chen et al. [61] also elucidated the potential application value of cinnamon essential oil and its components in alleviating oxidative stress. Zhang et al. [66] reported that the addition of clove extract substantially restrained carbonyl production in pork sausage, and this antioxidant activity of clove extract for restraining the carbonyl production might be due to the presence of phenolic compounds. Chauhan et al. [42] also suggested that Terminalia arjuna fruit extract significantly lowered the formation of protein carbonyls in raw pork in a control sample during storage. These conclusions are consistent with the findings of this study.

### 3.5. TVC Analysis

TVC serves as a critical indicator for assessing microbial contamination levels in meat products and is a primary factor contributing to spoilage during storage. It must be controlled within specified acceptable limits to ensure food safety compliance. According to the China National Standard (SB/T 10482-2008) [67] “Quality and safety requirements for pre-prepared meat products,” the TVC of refrigerated pre-prepared products stored at 0–4 °C should not exceed 6 lgCFU/g, with samples deemed spoiled if surpassing this threshold. As shown in Figure 4G–I, microbial proliferation was initially slow across all samples, but prefabricated bovine by-products exhibited significantly increased TVC (*p* < 0.05) during prolonged storage, with initial values (Day 0) of approximately 0.92, 0.94, and 0.73 lgCFU/g for liver, heart, and rumen samples, respectively. Notably, the rosemary extract-treated groups maintained significantly lower TVC (*p* < 0.05) than the controls throughout refrigeration, demonstrating superior antibacterial efficacy compared to cinnamon and nutmeg extracts. Specifically, in liver samples, accelerated microbial proliferation during days 7–11 led to control TVC reaching 6.06 lgCFU/g (exceeding limits) by day 12, whereas rosemary-treated samples remained compliant at 5.50 lgCFU/g, significantly lower than both controls and other treatment groups (5.93 and 5.92 lgCFU/g). Similarly, in heart and rumen samples exhibiting accelerated growth during days 7–13, control groups surpassed safety thresholds (6.06 and 6.05 lgCFU/g) by day 15, while rosemary-treated groups consistently maintained the lowest TVC below regulatory limits. This indicates that while rosemary, cinnamon, and nutmeg extracts partially inhibited microbial growth in bovine by-products during storage, rosemary extract demonstrated the most significant antimicrobial preservation effect, effectively extending the shelf life, consistent with the findings of Hernández-Hernández et al. [63], and attributable to its high carnosic acid content. Mechanistically, rosemary’s potent antibacterial action involves: (1) membrane disruption via hydrophobic carnosic acid and carnosol embedding into microbial phospholipid bilayers, increasing permeability and causing ion imbalance/intracellular leakage [59]; (2) enzyme inhibition through phenolic compounds binding sulfhydryl groups (-SH) of bacterial proteases (e.g., ATPase), disrupting energy metabolism [17]; and (3) quorum sensing interference by rosmarinic acid inhibiting acyl-homoserine lactone (AHL) signaling in Gram-negative bacteria, suppressing biofilm formation [68]. Notably, the moderate activity of cinnamon extract in this study contrasts with its strong antibacterial effects in lamb studies [17], highlighting the context-dependent functional differences in the antimicrobial efficacy of natural extracts.

### 3.6. Study Limitations

While this study provides valuable insights into optimizing marinating processes and enhancing storage stability of bovine by-products, several limitations should be acknowledged. First, microbial analysis was confined to TVC without evaluating specific spoilage microorganisms or pathogenic bacteria (e.g., Salmonella and Listeria), which may underestimate comprehensive microbial risks. Second, the orthogonal experimental design efficiently optimized marinating formulations but did not incorporate repeated measures for time-dependent variables (e.g., storage stability parameters), potentially affecting the robustness of longitudinal data interpretation. Third, the selection of plant extracts (rosemary, cinnamon, nutmeg, and clove) was based on preliminary antioxidant assays; however, other potent natural antioxidants (e.g., green tea polyphenols and oregano extract) were not comparatively evaluated, limiting the scope of antioxidant efficacy assessment. Future studies should address these limitations by integrating multi-omics approaches for microbial profiling, employing repeated-measures statistical designs, and screening broader antioxidant sources to refine their practical applications.

## 4. Conclusions

By innovatively combining orthogonal experimental design with natural antioxidant integration, this study establishes a technologically advanced framework for transforming bovine by-products (liver, heart, and rumen) into high-value foods, overcoming the key limitations of conventional methods. Using an L9 orthogonal array design, the research optimized marinating agents formulations and incorporated natural antioxidants (rosemary, cinnamon, and nutmeg extracts) to mitigate lipid oxidation and microbial spoilage during refrigerated storage. Results indicated that rosemary extract (0.3 g/kg) demonstrated superior performance, significantly reducing TBARS values (below 0.5 mg/kg) and carbonyl content while maintaining sensory scores above 55 throughout refrigeration. The optimized marinating formulations improved the texture, yield, and color stability, thereby extending the shelf life. Notably, rosemary extract excelled in preserving microbial and sensory qualities, with sensory scores of 56.67 (bovine liver), 55.67 (bovine heart), and 59.33 (bovine rumen), significantly surpassing those of the control group (*p* < 0.05). While natural extracts showed limited efficacy in color preservation, they effectively mitigated texture degradation, particularly in terms of maintaining chewiness. These findings underscore the potential of tailored marinating technologies and natural antioxidants—especially rosemary extract—to transform bovine by-products into high-value foods, aligning with consumer demand for natural preservatives and advancing sustainable practices in the livestock industry. This study offers actionable strategies for enhancing product quality, prolonging shelf life, and addressing economic and environmental challenges.

## Figures and Tables

**Table 1:** Sensory scoring standards for bovine by-products.

Evaluation Projects	Evaluation Criteria	Score
Texture (20)	Firm and elastic, with rapid recovery from finger indentation	15–20
	Slightly elastic, with slower recovery from finger indentation	8–14
	Slightly elastic, with slower recovery from finger indentation	0–7
Tissue state (20)	Fine texture, intact shape, smooth surface	15–20
	Somewhat rough texture, muscles not tightly packed, locally loose	8–14
	Rough texture, rough surface, large cracks, loose muscle tissue	0–7
Color (20)	Glossy surface, even color	15–20
	Indicates poor luster and average color	8–14
	No luster on the surface, poor color	0–7
Flavor (20)	The overall flavor is intense, with almost no fishy flavor	15–20
	The overall flavor is light, slightly fishy	8–14
	The overall flavor is single, fishy taste is very heavy	0–7
Overall acceptability (20)	Uniform color, strong aroma, overall good impression	15–20
	Relatively uniform color, weaker aroma, generally average impression	8–14
	Uneven color, off-flavors present, overall poor impression	0–7

**Table 2:** Analysis of differences in the basic nutrient content of prepared bovine by-products with the addition of different natural plant extracts.

By-products	Treatment	Protein Content (g/100 g)	Fat Content (g/100 g)	Moisture Content (g/100 g)	Ash Content (g/100 g)
Bovine liver	NG-XX	21.62 ± 0.03 ^a^	3.96 ± 0.07 ^a^	68.42 ± 0.50 ^a^	1.23 ± 0.07 ^a^
	NG-CK	19.35 ± 0.50 ^b^	3.97 ± 0.01 ^a^	70.26 ± 0.29 ^a^	1.23 ± 0.07 ^a^
	NG-MD	20.42 ± 0.19 ^b^	4.03 ± 0.07 ^a^	68.77 ± 0.67 ^a^	1.18 ± 0.10 ^a^
	NG-RG	19.45 ± 0.49 ^b^	4.04 ± 0.07 ^a^	69.19 ± 0.88 ^a^	1.17 ± 0.05 ^a^
	NG-RDK	20.48 ± 0.22 ^b^	4.17 ± 0.07 ^a^	69.25 ± 0.60 ^a^	1.19 ± 0.08 ^a^
Bovine heart	NX-XX	19.60 ± 0.31 ^a^	3.98 ± 0.08 ^a^	67.91 ± 1.11 ^a^	1.22 ± 0.14 ^a^
	NX-CK	17.88 ± 0.29 ^b^	4.00 ± 0.06 ^a^	67.76 ± 1.58 ^a^	1.15 ± 0.07 ^a^
	NX-MD	18.68 ± 0.61 ^ab^	3.92 ± 0.06 ^a^	67.93 ± 0.85 ^a^	1.12 ± 0.12 ^a^
	NX-RG	18.44 ± 0.18 ^b^	3.90 ± 0.12 ^a^	68.00 ± 1.43 ^a^	1.10 ± 0.11 ^a^
	NX-RDK	17.50 ± 0.16 ^b^	3.84 ± 0.19 ^a^	67.92 ± 1.68 ^a^	1.16 ± 0.06 ^a^
Bovine rumen	ND-XX	13.87 ± 0.17 ^ab^	3.30 ± 0.06 ^a^	79.17 ± 1.35 ^a^	1.11 ± 0.08 ^a^
	ND-CK	14.97 ± 0.46 ^a^	3.32 ± 0.07 ^a^	77.93 ± 1.58 ^a^	1.09 ± 0.13 ^a^
	ND-MD	15.43 ± 0.33 ^a^	3.18 ± 0.09 ^a^	76.62 ± 2.64 ^a^	1.15 ± 0.09 ^a^
	ND-RG	15.04 ± 0.59 ^a^	3.29 ± 0.14 ^a^	77.34 ± 3.02 ^a^	1.10 ± 0.08 ^a^
	ND-RDK	13.28 ± 0.67 ^b^	3.23 ± 0.02 ^a^	78.99 ± 1.33 ^a^	1.16 ± 0.10 ^a^

Note: XX: fresh sample, CK: basic marinating, MD: basic marinating + rosemary extract, RG: basic marinating + cinnamon extract, RDK: basic marinating + nutmeg extract. All values are expressed as the mean ± SD of three replicates. Means in the same column with different superscripts differ significantly: *p* < 0.05.

## Data Availability

The original contributions presented in the study are included in the article/Supplementary Material, further inquiries can be directed to the corresponding author.

## Supplementary Materials

The following supporting information can be downloaded at: https://www.mdpi.com/article/10.3390/foods14173036/s1, Figure S1: Sensory scores of bovine by-products with different (A) salt, (B) sugar, (C) monosodium glutamate, (D) pepper powder, (E) ginger powder, (F) cooking wine, (G) soy sauce, and (H) onion addition amount; Table S1: Experimental design of marination with different base marinade; Table S2: Factors and levels of orthogonal for bovine by-products; Table S3: Optimization of orthogonal experimental design for spices in marinated bovine liver; Table S4: Analysis of ANOVA for the orthogonal experiment on spices in marinated bovine liver; Table S5: Optimization of orthogonal experimental design for spices in marinated bovine heart; Table S6: Analysis of ANOVA for the orthogonal experiment on spices in marinated bovine heart; Table S7: Optimization of orthogonal experimental design for spices in marinated bovine rumen; Table S8: Analysis of ANOVA for the orthogonal experiment on spices in marinated bovine rumen.

## References

[1] Zhao Y., Zhang M., Law C.L., Yang C. (2024). New technologies and products for livestock and poultry bone processing: Research progress and application prospects: A review. Trends Food Sci. Technol..

[2] Mariutti L.R., Bragagnolo N. (2017). Influence of salt on lipid oxidation in meat and seafood products: A review. Food Res. Int..

[3] Hao S.Q., Du J.L., Sun X.Y., Hu G.H., Sun E.K., Li X.T., Jin Y., Zhao L.H. (2024). Characteristic flavor analysis of inner mongolia air-dried meat and the impact of vacuum tumbling curing on flavor. J. Food Biochem..

[4] Zhou Y., Hu M., Wang L. (2022). Effects of different curing methods on edible quality and myofibrillar protein characteristics of pork. Food Chem..

[5] Kumar Y., Yadav D.N., Ahmad T., Narsaiah K. (2015). Recent trends in the use of natural antioxidants for meat and meat products. Compr. Rev. Food Sci. Food Saf..

[6] Fan X.J., Liu S.Z., Li H., He J., Feng J.T., Zhang X., Yan H. (2019). Effects of *Portulaca oleracea L*. extract on lipid oxidation and color of pork meat during refrigerated storage. Meat Sci..

[7] Estévez M. (2021). Critical overview of the use of plant antioxidants in the meat industry: Opportunities, innovative applications and future perspectives. Meat Sci..

[8] Petcu C.D., Mihai O.D., Tăpăloagă D., Gheorghe-Irimia R.-A., Pogurschi E.N., Militaru M., Borda C., Ghimpeteanu O.-M. (2023). Effects of plant-based antioxidants in animal diets and meat products: A review. Foods.

[9] Malik A., Najda A., Bains A., Nurzyńska-Wierdak R., Chawla P. (2021). Characterization of citrus nobilis peel methanolic extract for antioxidant, antimicrobial, and anti-inflammatory activity. Molecules.

[10] Yin Y., Xing L.J., Zhou G.H., Zhang W.G. (2016). Antioxidative and antibacterial activities of rosemary extract in raw ground pork patties. J. Food Nutr. Res..

[11] Rajeev P.S., Johannah N.M., Gopakumar G., Maliakel B., Krishnakumar I.M. (2017). Optimization of antioxidant efficacy of a deflavored and decolorized rosemary extract: Effect of carnosol content on the oxidative stability of paprika colored beef patties. J. Food Sci. Technol..

[12] Yeddes M., Rybak K., Rebey I.B., Pietrzak D., Adamczak L., Hammami M., Wannes W.A., Witrowarajchert D., Tounsi M.S., Tixier A.S.F. (2025). Lipid oxidation and barrier properties of the coated freeze-dried chicken meat with gelatin-chitosan film enriched with rosemary (*Rosmarinus officinalis* L.) extract. Foods.

[13] Sirocchi V., Devlieghere F., Peelman N., Sagratini G., Maggi F., Vittori S., Ragaert P. (2017). Effect of Rosmarinus officinalis L. essential oil combined with different packaging conditions to extend the shelf life of refrigerated beef meat. Food Chem..

[14] Parvin R., Seo J.K., Eom J.U., Ahamed Z., Yang H.S. (2023). Inhibitory and antioxidative capacity of nutmeg extracts on reduction of lipid oxidation and heterocyclic amines in pan-roasted beef patties. Meat Sci..

[15] Moirangthem S., Patra G., Biswas S., Das A., Nath S., Verma A.K., Pal S., Chatterjee N., Bandyopadhyay S., Nanda P.K. (2024). Effect of nutmeg (*Myristica fragrans*) and tea tree (*Melaleuca alternifolia*) essential oils on the oxidative and microbial stability of chicken fillets during refrigerated storage. Foods.

[16] Shoqairan Y.I., Darwish H.K., Hamami M.A.H., Al-Juhaimi F.Y., Ahmed I.A.M., Babiker E.E. (2023). The influence of cinnamon powder on the antioxidant and antimicrobial properties of beef burger during refrigerated storage. LWT Food Sci. Technol..

[17] Hussain Z., Li X., Zhang D., Hou C., Ijaz M., Bai Y., Xiao X., Zheng X. (2021). Influence of adding cinnamon bark oil on meat quality of ground lamb during storage at 4 °C. Meat Sci..

[18] Zahid M.A., Choi J.Y., Seo J.K., Parvin R., Ko J., Yang H.S. (2020). Effects of clove extract on oxidative stability and sensory attributes in cooked beef patties at refrigerated storage. Meat Sci..

[19] Chen L., Kang Y.H. (2014). Antioxidant and enzyme inhibitory activities of Plebeian Herba (*Salvia plebeia* R. Br.) under different cultivation conditions. J. Agric. Food Chem..

[20] Ghasemian S.O., Ahmadi-Dastgerdi A., Abdollahi A., Tirtashi F.E., Zokaei M., Fallah N., NajafAbadi P.I., Dolatyari F., Verma A.K. (2024). The Effect of active packaging film based on chitosan containing rosemary (*Rosmarinus officinalis* L.) extract on cheese shelf life. J. Food Biochem..

[21] Gu F.L., Abbas S., Zhang X.M. (2009). Optimization of Maillard reaction products from casein–glucose using response surface methodology. LWT Food Sci. Technol..

[22] (2016). Determination of Moisture Content in Food.

[23] (2016). Determination of Ash Content in Food.

[24] (2016). Determination of Protein Content in Food.

[25] (2016). Determination of Fat Content in Food.

[26] (2008). Criterion for Sensory Evaluation of Meat and Meat Products.

[27] Tan C., Li X., Yu Y., Nie S., Wen Q., Tu Z., Zhang L. (2024). Effects of five thermal processing methods on the physicochemical properties and flavor characteristics of grass carp meat. LWT Food Sci. Technol..

[28] (2022). Food Microbiological Examination—Determination of Total Plate Count.

[29] Ballantyne K.N., van Oorschot R.A., Mitchell R.J. (2008). Reduce optimisation time and effort: Taguchi experimental design methods. Forensic Sci. Int. Genet..

[30] Fu R., Zhang Y., Guo Y., Liu F., Chen F. (2014). Determination of phenolic contents and antioxidant activities of extracts of *Jatropha curcas* L. seed shell, a by-product, a new source of natural antioxidant. Ind. Crops Prod..

[31] Ghorbani A., Mahmoudifar K., Shokri S., Mazaheri Y., Shamloo E., Rezagholizade-shirvan A., Elhamirad A.H. (2024). Effect of Allium Jesdianum’s extract on the physicochemical, antioxidant, antimicrobial and sensory properties of sausage characteristics. Food Chem. X.

[32] Li Y., Jiang B., Zhang T., Mu W., Liu J. (2008). Antioxidant and free radical-scavenging activities of chickpea protein hydrolysate (CPH). Food Chem..

[33] Huang M., Wang H., Xu X., Lu X., Song X., Zhou G. (2020). Effects of nanoemulsion-based edible coatings with composite mixture of rosemary extract and ε-poly-L-lysine on the shelf life of ready-to-eat carbonado chicken. Food Hydrocoll..

[34] Sun Y., Zhang M., Bhandari B., Bai B. (2021). Nanoemulsion-based edible coatings loaded with fennel essential oil/cinnamaldehyde: Characterization, antimicrobial property and advantages in pork meat patties application. Food Control.

[35] Wang Z.F., He Z.F., Zhang D., Li H.J., Wang Z.M. (2020). Using oxidation kinetic models to predict the quality indices of rabbit meat under different storage temperatures. Meat Sci..

[36] (2024). Standard for Use of Food Addditives.

[37] Tomasevic I., Djekic I., Furnol M.F.I., Terjung N., Lorenzo J.M. (2021). Recent advances in meat color research. Curr. Opin. Food Sci..

[38] Faustman C., Cassens R.G. (1990). The biochemical basis for discoloration in fresh meat: A review. J. Muscle Foods.

[39] Li X., Zhang Y., Li Z., Li M., Liu Y., Zhang D. (2017). The effect of temperature in the range of −0.8 to 4 °C on lamb meat color stability. Meat Sci..

[40] Wei Z.X., Zhang J.J., Zhang H.C., Zhang N., Zhang R.Y., Li L.J., Liu G.Q. (2021). Effect of nanoemulsion loading a mixture of clove essential oil and carboxymethyl chitosan-coated ε-polylysine on the preservation of donkey meat during refrigerated storage. J. Food Process. Preserv..

[41] Sujiwo J., Kim D., Jang A. (2018). Relation among quality traits of chicken breast meat during cold storage: Correlations between freshness traits and torrymeter values. Poult. Sci..

[42] Chauhan P., Pradhan S.R., Das A., Nanda P.K., Bandyopadhyay N., Das A.K. (2019). Inhibition of lipid and protein oxidation in raw ground pork by Terminalia arjuna fruit extract during refrigerated storage. Asian Australas. J. Anim. Sci..

[43] Vaz-Pires P., Seixas P.P., Mota M., Lapa-Guimarães J., Pickova J., Lindo A., Silva T. (2008). Sensory, microbiological, physical and chemical properties of cuttlefish (*Sepia officinalis*) and broadtail shortfin squid (*Illex coindetii*) stored in ice. LWT Food Sci. Technol..

[44] Chaijan M., Benjakul S., Visessanguan W., Faustman C. (2005). Changes of pigments and color in sardine (*Sardinella gibbosa*) and mackerel (Rastrelliger kanagurta) muscle during iced storage. Food Chem..

[45] Jiang Z.Y., Woollard A.C.S., Wolff S.P. (1991). Lipid hydroperoxide measurement by oxidation of Fe^2+^ in the presence of xylenol orange. comparison with the TBA assay and an lodometric method. Lipid.

[46] Van Buren J.B., Epperson B., Jepsen S., Heimbuch M., Oliver K., Nasados J., Bass P.D., Colle M.J. (2024). Acerola cherry and rosemary extracts improve color and delay lipid oxidation in previously frozen beef. Foods.

[47] Szmańko T., Lesiów T., Górecka J. (2021). The water-holding capacity of meat: A reference analytical method. Food Chem..

[48] Li N., Xie J., Chu Y.M. (2023). Degradation and evaluation of myofibril proteins induced by endogenous protease in aquatic products during storage: A review. Food Sci. Biotechnol..

[49] Liu J., Arner A., Puolanne E., Ertbjerg P. (2016). On the water-holding of myofibrils: Effect of sarcoplasmic protein denaturation. Meat Sci..

[50] Colle M.C., Richard R.P., Smith D.M., Colle M.J., Loucks W.I., Gray S.J., Reynolds Z.D., Sutton H.A., Nasados J.A., Doumit M.E. (2019). Dry potato extracts improve water holding capacity, shelf life, and sensory characteristics of fresh and precooked beef patties. Meat Sci..

[51] Shariatmadari F., Ahmadi H. (2025). An overview of rosemary in modern poultry nutrition and production. World’s Poult. Sci. J..

[52] Zhuang X., Han M., Bai Y., Liu Y., Xing L., Xu X.L., Zhou G.H. (2018). Insight into the mechanism of myofibrillar protein gel improved by insoluble dietary fiber. Food Hydrocoll..

[53] Li F., Zhong Q., Kong B., Wang B., Pan N., Xia X. (2020). Deterioration in quality of quick-frozen pork patties induced by changes in protein structure and lipid and protein oxidation during frozen storage. Food Res. Int..

[54] Xiao X.C., Lin D., Cao K.Y., Sun L.C., Chen Y.L., Weng L., Zhang L.J., Cao M.J. (2023). Properties of Pacific white shrimp (*Litopenaeus vannamei*) collagen and its degradation by endogenous proteinases during cold storage. Food Chem..

[55] Cáceres E., García M.L., Selgas M.D. (2008). Effect of pre-emulsified fish oil-as source of PUFA n-3- on microstructure and sensory properties of *mortadella*, a Spanish bologna-type sausage. Meat Sci..

[56] Hajlaoui H., Arraouadi S., Mighri H., Chaaibia M., Gharsallah N., Ros G., Nieto G., Kadri A. (2019). Phytochemical constituents and antioxidant activity of *Oudneya Africana L.* leaves extracts: Evaluation effects on fatty acids and proteins oxidation of beef burger during refrigerated storage. Antioxidants.

[57] Karabagias I., Badeka A., Kontominas M.G. (2011). Shelf life extension of lamb meat using thyme or oregano essential oils and modified atmosphere packaging. Meat Sci..

[58] Babaolu A.S., Poan H.B., Ainiwaer T., Zkan H., Mutlu E.K., Karakaya M. (2023). Assessment of garlic and onion powder as natural antioxidant on the physicochemical properties, lipid-protein oxidation and sensorial characteristics of beef and chicken during frozen storage. J. Food Saf. Food Qual..

[59] Bellucci E.R.B., Munekata P.E.S., Pateiro M., Lorenzo J.M., da Silva Barretto A.C. (2021). Red pitaya extract as natural antioxidant in pork patties with total replacement of animal fat. Meat Sci..

[60] Hernández-Hernández E., Ponce-Alquicira E., Jaramillo-Flores M.E., Guerrero Legarreta I. (2009). Antioxidant effect rosemary (*Rosmarinus officinalis* L.) and oregano (*Origanum vulgare* L.) extracts on TBARS and colour of model raw pork batters. Meat Sci..

[61] Chen X.H., Shang S.F., Yan F., Jiang H., Zhao G.J., Tian S., Chen R., Chen D.J., Dang Y.F. (2023). Antioxidant activities of essential oils and their major components in scavenging free radicals, inhibiting lipid oxidation and reducing cellular oxidative stress. Molecules.

[62] Brewer M.S. (2011). Natural antioxidants: Sources, compounds, mechanisms of action, and potential applications. Compr. Rev. Food Sci. Food Saf..

[63] Wang C., Wang Y., Song Y., Ren M., Gao Z., Ren J. (2024). Effect of onion skin powder on color, lipid, and protein oxidative stability of premade beef patty during cold storage. Sci. Rep..

[64] Mtibaa A.C., Smaoui S., Ben Hlima H., Sellem I., Ennouri K., Mellouli L. (2019). Enterocin BacFL31 from a safety *Enterococcus faecium* FL31: Natural preservative agent used alone and in combination with aqueous peel onion (*Allium cepa*) extract in ground beef meat storage. BioMed Res. Int..

[65] Lee M.A., Kim T.K., Hwang K.E., Choi Y.J., Park S.H., Kim C.J., Choi Y.S. (2019). Kimchi extracts as inhibitors of colour deterioration and lipid oxidation in raw ground pork meat during refrigerated storage. J. Sci. Food Agric..

[66] Zhang H., Peng X., Li X., Wu J., Guo X. (2017). The application of clove extract protects chinese-style sausages against oxidation and quality deterioration. Korean J. Food Sci. Anim. Resour..

[67] (2008). Quality Safety Requirement of Prepared Meat Products.

[68] Corral-Lugo A., Daddaoua A., Ortega A., Espinosa-Urgel M., Krell T. (2016). So different and still so similar: The plant compound rosmarinic acid mimics bacterial homoserine lactone quorum sensing signals. Commun. Integr. Biol..

