# Supplementary material for: Optimization of Marinating Process and Evaluation of Storage Stability in Bovine By-products
Source: Foods. 2025 Aug 29;14(17):3036. doi: 10.3390/foods14173036 (PMC12428361; doi:10.3390/foods14173036)
Supplement: Supplementary file 1 [file foods-14-03036-s001.zip › Figure S1.pdf]

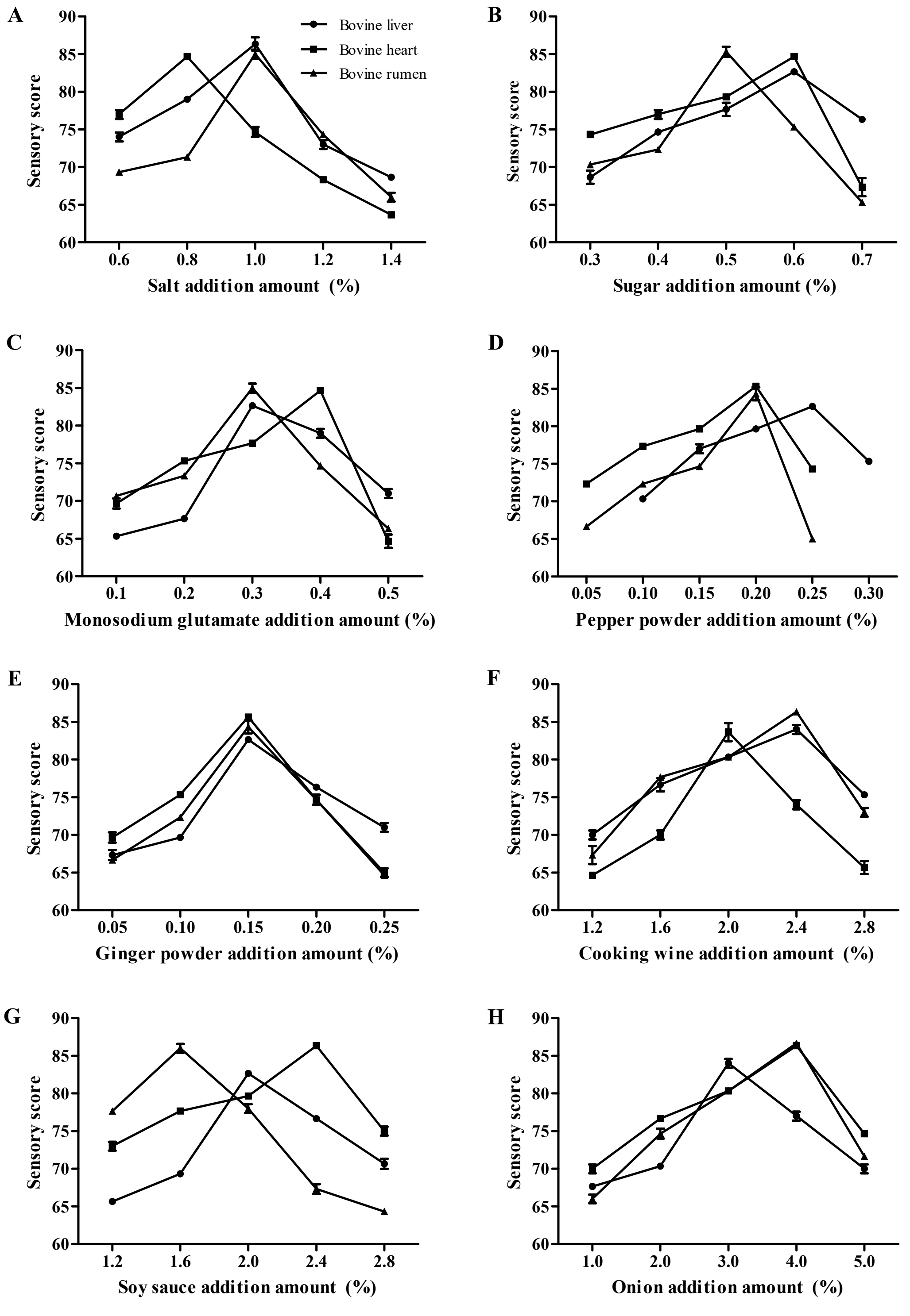

Figure S1 Sensory scores of bovine by-products with different (A) salt, (B) sugar, (C) monosodium glutamate, (D) pepper powder, (E) ginger powder, (F) cooking wine, (G) soy sauce, and (H) onion addition amount.
