# Supplementary material for: Optimization of Marinating Process and Evaluation of Storage Stability in Bovine By-products
Source: Foods. 2025 Aug 29;14(17):3036. doi: 10.3390/foods14173036 (PMC12428361; doi:10.3390/foods14173036)
Supplement: Supplementary file 1 [file foods-14-03036-s001.zip › Table S1.pdf]

Table S1 Experimental design of marination with different base marinade

Base marinade	Bovine liver	Bovine heart	Bovine rumen
NaCl	0.6%、0.8%、1.0%、1.2%、1.4%		
Sugar	0.3%、0.4%、0.5%、0.6%、0.7%		
Monosodium glutamate	0.1%、0.2%、0.3%、0.4%、0.5%		
Ginger powder	0.05%、0.1%、0.15%、0.2%、0.25%		
Pepper powder	0.05%、0.1%、0.15%、0.2%、0.25%		
Cooking wine	1.2%、1.6%、2.0%、2.4%、2.8%		
Soya sauce	1.2%、1.6%、2.0%、2.4%、2.8%		
Onion	1%、2%、3%、4%、5%		
