# Supplementary material for: Optimization of Marinating Process and Evaluation of Storage Stability in Bovine By-products
Source: Foods. 2025 Aug 29;14(17):3036. doi: 10.3390/foods14173036 (PMC12428361; doi:10.3390/foods14173036)
Supplement: Supplementary file 1 [file foods-14-03036-s001.zip › Table S2.pdf]

Table S2 Factors and levels of orthogonal for bovine by-products

Kinds	Level	Factor							
		NaCl%	Sugar /%	Monosodium glutamate/%	Ginger powder /%	Pepper powder /%	Cooking wine /%	Soya sauce/%	Onion /%
Bovine liver	1	0.8	0.5	0.2	0.1	0.2	2.0	1.6	2
	2	1.0	0.6	0.3	0.15	0.25	2.4	2.0	3
	3	1.2	0.7	0.4	0.2	0.3	2.8	2.4	4
Bovine heart	1	0.6	0.5	0.3	0.1	0.15	1.6	2.0	3
	2	0.8	0.6	0.4	0.15	0.20	2.0	2.4	4
	3	1.0	0.7	0.5	0.2	0.25	2.4	2.8	5
Bovine rumen	1	0.8	0.4	0.2	0.1	0.15	2.0	1.2	3
	2	1.0	0.5	0.3	0.15	0.20	2.4	1.6	4
	3	1.2	0.6	0.4	0.2	0.25	2.8	2.0	5
