# Supplementary material for: Optimization of Marinating Process and Evaluation of Storage Stability in Bovine By-products
Source: Foods. 2025 Aug 29;14(17):3036. doi: 10.3390/foods14173036 (PMC12428361; doi:10.3390/foods14173036)
Supplement: Supplementary file 1 [file foods-14-03036-s001.zip › Table S3.pdf]

Table S3 Optimization of orthogonal experimental design for spices in marinated bovine liver

No.	NaCl	Sugar	Monosodium glutamate	Ginger powder	Pepper powder	Cooking wine	Soya sauce	Onion	Sensory score
1	1	1	1	1	1	1	1	1	48.00
2	1	1	1	1	2	2	2	2	72.00
3	1	1	1	1	3	3	3	3	51.12
4	1	2	2	2	1	1	1	2	73.00
5	1	2	2	2	2	2	2	3	88.70
6	1	2	2	2	3	3	3	1	61.56
7	1	3	3	3	1	1	1	3	47.25
8	1	3	3	3	2	2	2	1	64.00
9	1	3	3	3	3	3	3	2	51.23
10	2	1	2	3	1	2	3	1	76.00
11	2	1	2	3	2	3	1	2	79.00
12	2	1	2	3	3	1	2	3	62.00
13	2	2	3	1	1	2	3	2	79.00
14	2	2	3	1	2	3	1	3	68.00
15	2	2	3	1	3	1	2	1	70.00
16	2	3	1	2	1	2	3	3	69.00
17	2	3	1	2	2	3	1	1	64.28
18	2	3	1	2	3	1	2	2	75.00
19	3	1	3	2	1	3	2	1	55.00
20	3	1	3	2	2	1	3	2	69.77
21	3	1	3	2	3	2	1	3	58.00
22	3	2	1	3	1	3	2	2	65.00
23	3	2	1	3	2	1	3	3	56.00
24	3	2	1	3	3	2	1	1	54.87
25	3	3	2	1	1	3	2	3	53.00
26	3	3	2	1	2	1	3	1	51.00
27	3	3	2	1	3	2	1	2	63.98
K_1	556.86	570.89	555.27	556.10	565.25	552.02	556.38	544.71	
K_2	642.28	616.13	608.24	614.31	612.75	625.55	604.70	627.98	
K_3	526.62	538.74	562.25	555.35	547.76	548.19	564.68	553.07	
k_1	185.62	190.30	185.09	185.37	188.42	184.01	185.46	181.57	
k_2	214.09	205.38	202.75	204.77	204.25	208.52	201.57	209.33	

k_3	175.54	179.58	187.42	185.12	182.59	182.73	188.23	184.36
R	38.55	25.80	17.66	19.65	21.66	25.79	16.11	27.76
Factor level order	A>H>B>F>E>D>C>G							
Optimal level	2	2	2	2	2	2	2	2
Optimal combination	$A_2H_2B_2F_2E_2D_2C_2G_2$							
