# Supplementary material for: Optimization of Marinating Process and Evaluation of Storage Stability in Bovine By-products
Source: Foods. 2025 Aug 29;14(17):3036. doi: 10.3390/foods14173036 (PMC12428361; doi:10.3390/foods14173036)
Supplement: Supplementary file 1 [file foods-14-03036-s001.zip › Table S4.pdf]

Table S4 Analysis of ANOVA for the orthogonal experiment on spices in marinated bovine liver

Source of variation	S.S.	DF.	M.S.	*F*-value	*p*-value	Sig.
Model	2863.148[a]	16	178.947	12.094	0.000	
Intercept	110305.466	1	110305.466	7454.654	0.000	
V_A	799.566	2	399.783	27.018	0.000	
V_B	335.907	2	167.954	11.351	0.003	*
V_C	184.060	2	92.030	6.220	0.018	*
V_D	254.268	2	127.134	8.592	0.007	*
V_E	251.328	2	125.664	8.493	0.007	*
V_F	422.441	2	211.220	14.275	0.001	*
V_G	148.345	2	74.172	5.013	0.031	*
V_H	467.233	2	233.616	15.788	0.001	*
Error	147.969	10	14.797			
Total	113316.582	27				
Revised total	3011.116	26				
R^2			0.951			

Note: A means NaCl; B means sugar; C means monosodium glutamate; D means Ginger powder; E means Pepper powder; F means Cooking wine; G means Soya sauce; H means Onion. S.S.: denotes sum. DF.: denotes degree of freedom. M.S.: denotes mean square. Sig.: indicates significance. “*” indicates significant difference ($p < 0.05$).
