# Supplementary material for: Optimization of Marinating Process and Evaluation of Storage Stability in Bovine By-products
Source: Foods. 2025 Aug 29;14(17):3036. doi: 10.3390/foods14173036 (PMC12428361; doi:10.3390/foods14173036)
Supplement: Supplementary file 1 [file foods-14-03036-s001.zip › Table S5.pdf]

Table S5 Optimization of orthogonal experimental design for spices in marinated bovine heart

No.	NaCl	Sugar	monosodium glutamate	Ginger powder	Pepper powder	Cooking wine	Soya sauce	Onion	Sensory score
1	1	1	1	1	1	1	1	1	45.70
2	1	1	1	1	2	2	2	2	70.25
3	1	1	1	1	3	3	3	3	42.00
4	1	2	2	2	1	1	1	2	78.58
5	1	2	2	2	2	2	2	3	93.00
6	1	2	2	2	3	3	3	1	62.00
7	1	3	3	3	1	1	1	3	49.20
8	1	3	3	3	2	2	2	1	68.00
9	1	3	3	3	3	3	3	2	57.40
10	2	1	2	3	1	2	3	1	71.00
11	2	1	2	3	2	3	1	2	78.80
12	2	1	2	3	3	1	2	3	65.00
13	2	2	3	1	1	2	3	2	76.40
14	2	2	3	1	2	3	1	3	76.90
15	2	2	3	1	3	1	2	1	63.20
16	2	3	1	2	1	2	3	3	59.60
17	2	3	1	2	2	3	1	1	74.10
18	2	3	1	2	3	1	2	2	71.10
19	3	1	3	2	1	3	2	1	57.50
20	3	1	3	2	2	1	3	2	71.40
21	3	1	3	2	3	2	1	3	59.20
22	3	2	1	3	1	3	2	2	73.70
23	3	2	1	3	2	1	3	3	52.00
24	3	2	1	3	3	2	1	1	63.10
25	3	3	2	1	1	3	2	3	48.90
26	3	3	2	1	2	1	3	1	51.90
27	3	3	2	1	3	2	1	2	64.20
K_1	566.13	560.85	551.55	539.45	560.58	548.08	589.78	556.50	
K_2	636.10	638.88	613.38	626.48	636.35	624.75	610.65	641.83	
K_3	541.90	544.40	579.20	578.20	547.20	571.30	543.70	545.80	
k_1	188.71	186.95	183.85	179.82	186.86	182.69	196.59	185.50	
k_2	212.03	212.96	204.46	208.83	212.12	208.25	203.55	213.94	

k_3	180.63	181.47	193.07	192.73	182.40	190.43	181.23	181.93
R	31.40	31.49	20.61	29.01	29.72	25.56	22.32	32.01
Factor level order	H>B>A>E>D>F>G>C							
Optimal level	2	2	2	2	2	2	2	2
Optimal combination	$H_2B_2A_2E_2D_2F_2G_2C_2$							
