# Supplementary material for: Optimization of Marinating Process and Evaluation of Storage Stability in Bovine By-products
Source: Foods. 2025 Aug 29;14(17):3036. doi: 10.3390/foods14173036 (PMC12428361; doi:10.3390/foods14173036)
Supplement: Supplementary file 1 [file foods-14-03036-s001.zip › Table S6.pdf]

Table S6 Analysis of ANOVA for the orthogonal experiment on spices in marinated bovine heart

Source of variation	S.S.	DF.	M.S.	*F*-value	*p*-value	Sig.
Model	3466.876[a]	16	216.68	10.189	0	
Intercept	112666.276	1	112666.276	5298.041	0	
V_A	531.723	2	265.862	12.502	0.002	*
V_B	566.139	2	283.07	13.311	0.002	*
V_C	213.176	2	106.588	5.012	0.031	*
V_D	422.472	2	211.236	9.933	0.004	*
V_E	513.624	2	256.812	12.076	0.002	*
V_F	343.495	2	171.747	8.076	0.008	*
V_G	260.786	2	130.393	6.132	0.018	*
V_H	615.461	2	307.731	14.471	0.001	*
Residual	212.656	10	21.266			
Total	116345.809	27				
Revised total	3679.533	26				
R^2			0.942			

Note: A means NaCl; B means sugar; C means monosodium glutamate; D means Ginger powder; E means Pepper powder; F means Cooking wine; G means Soya sauce; H means Onion. S.S.: denotes sum. DF.: denotes degree of freedom. M.S.: denotes mean square. Sig.: indicates significance. "*" indicates significant difference ($p < 0.05$).
