# Supplementary material for: Optimization of Marinating Process and Evaluation of Storage Stability in Bovine By-products
Source: Foods. 2025 Aug 29;14(17):3036. doi: 10.3390/foods14173036 (PMC12428361; doi:10.3390/foods14173036)
Supplement: Supplementary file 1 [file foods-14-03036-s001.zip › Table S7.pdf]

Table S7 Optimization of orthogonal experimental design for spices in marinated bovine rumen

No.	NaCl	Sugar	monosodium glutamate	Ginger powder	Pepper powder	Cooking wine	Soya sauce	Onion	Sensory score
1	1	1	1	1	1	1	1	1	48.20
2	1	1	1	1	2	2	2	2	71.90
3	1	1	1	1	3	3	3	3	45.00
4	1	2	2	2	1	1	1	2	74.58
5	1	2	2	2	2	2	2	3	93.20
6	1	2	2	2	3	3	3	1	62.00
7	1	3	3	3	1	1	1	3	54.00
8	1	3	3	3	2	2	2	1	65.70
9	1	3	3	3	3	3	3	2	57.00
10	2	1	2	3	1	2	3	1	74.20
11	2	1	2	3	2	3	1	2	78.00
12	2	1	2	3	3	1	2	3	60.12
13	2	2	3	1	1	2	3	2	75.25
14	2	2	3	1	2	3	1	3	69.60
15	2	2	3	1	3	1	2	1	65.00
16	2	3	1	2	1	2	3	3	62.25
17	2	3	1	2	2	3	1	1	71.42
18	2	3	1	2	3	1	2	2	75.40
19	3	1	3	2	1	3	2	1	54.20
20	3	1	3	2	2	1	3	2	66.20
21	3	1	3	2	3	2	1	3	50.80
22	3	2	1	3	1	3	2	2	68.80
23	3	2	1	3	2	1	3	3	56.00
24	3	2	1	3	3	2	1	1	53.62
25	3	3	2	1	1	3	2	3	57.30
26	3	3	2	1	2	1	3	1	59.00
27	3	3	2	1	3	2	1	2	62.80
K1	571.58	548.62	552.59	554.05	568.78	558.50	563.02	553.34	
K2	631.24	618.05	621.20	610.05	631.02	609.72	611.62	629.93	
K3	528.72	564.87	557.75	567.44	531.74	563.32	556.90	548.27	
k1	190.53	182.87	184.20	184.68	189.59	186.17	187.67	184.45	
k2	210.41	206.02	207.07	203.35	210.34	203.24	203.87	209.98	

k3	176.24	188.29	185.92	189.15	177.25	187.77	185.63	182.76
R	34.17	23.14	22.87	18.67	33.09	17.07	18.24	27.22
Factor level order	A>E>H>B>C>D>G>F							
Optimal level	2	2	2	2	2	2	2	2
Optimal combination	$A_2E_2H_2B_2C_2D_2G_2F_2$							
