# Supplementary material for: Optimization of Marinating Process and Evaluation of Storage Stability in Bovine By-products
Source: Foods. 2025 Aug 29;14(17):3036. doi: 10.3390/foods14173036 (PMC12428361; doi:10.3390/foods14173036)
Supplement: Supplementary file 1 [file foods-14-03036-s001.zip › Table S8.pdf]

Table S8 Analysis of ANOVA for the orthogonal experiment on spices in marinated bovine rumen

Source of variation	S.S.	DF.	M.S.	*F*-value	*p*-value	Sig.
Model	2798.736a	16	174.921	8.85	0.001	
Intercept	111045.584	1	111045.584	5618.392	0	
V_A	589.135	2	294.568	14.904	0.001	*
V_B	293.063	2	146.531	7.414	0.011	*
V_C	324.439	2	162.22	8.208	0.008	*
V_D	190.033	2	95.017	4.807	0.034	*
V_E	559.344	2	279.672	14.15	0.001	*
V_F	177.766	2	88.883	4.497	0.04	*
V_G	199.766	2	99.883	5.054	0.03	*
V_H	465.188	2	232.594	11.768	0.002	*
Residual	197.647	10	19.765			
Total	114041.967	27				
Revised total	2996.382	26				
R^2			0.934			

Note: A means NaCl; B means sugar; C means monosodium glutamate; D means Ginger powder; E means Pepper powder; F means Cooking wine; G means Soya sauce; H means Onion. S.S.: denotes sum. DF.: denotes degree of freedom. M.S.: denotes mean square. Sig.: indicates significance. “*” indicates significant difference ($p < 0.05$).
